# Hydration Characteristics of Low-Heat Cement Substituted by Fly Ash and Limestone Powder

**DOI:** 10.3390/ma8095277

**Published:** 2015-09-01

**Authors:** Si-Jun Kim, Keun-Hyeok Yang, Gyu-Don Moon

**Affiliations:** 1 Department of Plant Architectural Engineering, Kyonggi University, Suwon, Kyonggi-do 16227, Korea; E-Mail: season@kgu.ac.kr; 2 Department of Architectural Engineering, Kyonggi University, Graduate School, Suwon, Kyonggi-do 16227, Korea; E-Mail: mgd0123@kcl.re.kr

**Keywords:** low-heat cement, fly ash, limestone powder, heat of hydration, pore size

## Abstract

This study proposed a new binder as an alternative to conventional cement to reduce the heat of hydration in mass concrete elements. As a main cementitious material, low-heat cement (LHC) was considered, and then fly ash (FA), modified FA (MFA) by vibrator mill, and limestone powder (LP) were used as a partial replacement of LHC. The addition of FA delayed the induction period at the hydration heat curve and the maximum heat flow value (*q*_max_) increased compared with the LHC based binder. As the proportion and fineness of the FA increased, the induction period of the hydration heat curve was extended, and the *q*_max_ increased. The hydration production of Ca(OH)_2_ was independent of the addition of FA or MFA up to an age of 7 days, beyond which the amount of Ca(OH)_2_ gradually decreased owing to their pozzolanic reaction. In the case of LP being used as a supplementary cementitious material, the induction period of the hydration heat curve was reduced by comparison with the case of LHC based binder, and monocarboaluminate was observed as a hydration product. The average pore size measured at an age of 28 days was smaller for LHC with FA or MFA than for 100% LHC.

## 1. Introduction

Cement, which is widely used as a construction material, forms hydrates such as calcium silicate hydrate (C–S–H), ettringite, and Ca(OH)_2_ through a hydration reaction in which hydration heat is produced within the concrete because of an exothermic reaction. Since the thermal cracking of concrete reduces its internal force, watertightness, and durability, an appropriate measure is required to control the heat of hydration. The factors that influence the hydration heat of concrete include the placement temperature of the concrete and the hydration heat characteristics of the cement (hydration heat of cement, hydration reaction rate of cement, and unit cement amount). 

In fields involving the construction of mass concrete structures, a pre-cooling method, which lowers the temperature of the aggregate and mixing water, and a post-cooling method, which employs cooling pipes, is currently used to control the hydration heat of concrete. With respect to the material, efforts are also being directed toward lowering the hydration heat of concrete. A typical method involves reducing the use of cement by substituting ordinary Portland cement (OPC) with a mineral admixture, such as fly ash (FA) or ground-granulated blast-furnace slag (GGBS). Because of its reduced OPC usage, concrete that uses a mineral admixture as a supplementary cementitious material (SCM) for cement exhibits not only a low hydration heat but also a reduction in its greenhouse-gas emissions [1,2,3,4]. Thus, various studies have been conducted on blended cement that uses a mineral admixture as SCM for cement (binary or ternary blended cement). However, carbonation and low early strength have been identified as problems for blended cement with a large amount of mineral admixture. In addition to the above method which involves the substitution of the cement with a mineral admixture such as FA or GGBS, low-heat cement (LHC), which has a relatively low hydration heat compared with OPC, can be also used to reduce the hydration heat of the concrete. The hydration heats of the cement components, tricalcium silicate (C_3_S), dicalcium silicate (C_2_S), tricalcium aluminate (C_3_A) and tetracalcium aluminoferrite (C_4_AF) are 510, 247, 1356 and 427 J/g, respectively, and the final hydration heat of the cement is directly affected by the proportion of these components [5]. According to ASTM C 150 [6], the hydration heat of LHC (type IV) is defined as 250 J/g or less, and the C_3_S, C_2_S and C_3_A contents are defined as less than or equal to 35%, 40% and 7%, respectively. According to European standards EN 197-1 [7] and EN 14,216 [8], LHCs are categorized into low-heat common cement and very-low-heat special cement, and their hydration heats are defined to be less than or equal to 270 and 220 J/g, respectively. Studies have been conducted on the hydration reaction and strength-development properties of LHC, but these are scarce compared with the studies on blended cement [9,10]. The emergence of mega-concrete structures has generated demand for ultra-low-heat cement, which has a lower hydration heat than conventional blended cement and LHC [9]. Low-heat blended cement, which employs a mineral admixture such as FA in place of LHC, has a lower hydration heat than OPC, and has also attracted much attention [11].

This study aims to analyze the hydration rate, heat of hydration, hydration products, and pore characteristics of binders that use FA and limestone powder (LP) as supplementary cementitious materials of LHC.

## 2. Materials and Methods

### 2.1. Materials

**Figure 1:**
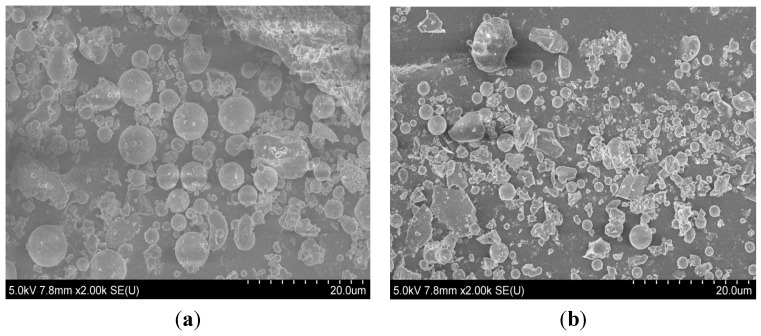
Scanning electron microscope (SEM) images of (**a**) fly ash (FA) and (**b**) modified FA (MFA) with particle size of 20 μm.

Table 1 shows the chemical and Bogue composition of the LHC, FA and LP by ASTM C 114 [12]. The C_3_S, C_2_S, C_3_A and C_4_AF contents of the LHC are 31%, 48%, 3% and 11%, respectively. The C_3_S and C_3_A contents are lower than the OPC content, whereas the C_2_S and C_4_AF contents are higher. The CaO and (SiO_2_ + Al_2_O_3_ + Fe_2_O_3_) contents of the FA are 3.8% and 91.4%, respectively, which corresponds to “Class C” of ASTM C 618 [13]. Table 2 shows physical characteristics which are specific gravity and blain of the LHC, FA, and LP by ASTM C 188 [14] and ASTM C 204 [15]. Figure 1 shows a scanning electron microscope (SEM) image of modified fly ash (MFA), which is FA that has been altered using a vibratory mill. The FA particles are spherical, whereas the particles of the MFA are mostly irregular in shape. 

**Table 1:** Chemical and Bogue composition of raw materials (by weight, %).

Materials	Chemical Composition										Bogue Composition			
	SiO_2_	Al_2_O_3_	Fe_2_O_3_	CaO	MgO	K_2_O	Na_2_O	TiO_2_	SO_3_	LOI *	C_3_S	C_2_S	C_3_A	C_4_AF
LHC	25.3	3.1	3.4	62.5	1.7	0.57	0.10	0.09	1.9	0.8	31	48	3	11
FA	64.0	21.9	5.5	3.8	1.2	1.1	1.0	1.5	-	2.23	-	-	-	-
LP	17.7	8.2	0.6	47.5	2.1	-	-	-	0.3	22.3	-	-	-	-

LHC: low-heat cement; FA: fly ash; LP: limestone powder; * Loss on ignition.

**Table 2:** Physical characteristics of raw materials.

Materials	Specific Gravity	Blaine (cm^2^/g)
LHC	3.18	3440
FA	2.25	3520
MFA	2.27	5510
LP	2.81	3420

MFA: modified fly ash.

### 2.2. Preparation of Specimens

As binders, LHC and supplementary cementitious materials which are FA, LP, and a mixture of FA and LP were used. There were nine types of binder, including LH100, which employed 100% LHC. The binder proportions are presented in Table 3. 

To analyze the effect of the fineness and amount of FA substitute on the hydration of the LHC, 20 wt % of the LHC was replaced by FA (LH80FA20), and 10, 20 and 30 wt % of the LHC was replaced by MFA (LH90MFA10, LH80MFA20 and LH70MFA30). The LP replaced 5, 10 and 15 wt % of the LHC (LH95LP5, LH90LP10 and LH85LP5). Additionally, a mixture of 15% FA and 5% LP was used as a binder (LH80MFA15LP5) to replace 20% of the LHC.

**Table 3:** Binder proportions (by weight, %).

Binder	LHC	FA	MFA	LP
LH100	100	-	-	-
LH80FA20	80	20	-	-
LH90MFA10	90	-	10	-
LH80MFA20	80	-	20	-
LH70MFA30	70	-	30	-
LH95LP5	95	-	-	5
LH90LP10	90	-	-	10
LH85LP15	85	-	-	15
LH80MFA15LP5	80	-	15	5

Pastes were used as specimens for the thermogravimetric analysis (TGA), X-ray diffraction (XRD), and mercury intrusion porosimetry (MIP) measurements. When fabricating the pastes, the *w*/*b* (water/binder ratio) was set as 0.5. Following their formation, the pastes were cured in a chamber at a constant temperature and humidity of 23 °C and 95% relative humidity (RH), respectively, for the desired aging time, and subsequently dipped in isopropanol. All the specimens were dried in a vacuum dryer at a temperature of 40 °C for one day before the measurements were performed. The pastes were formed into a powder to use as specimens for the TGA and XRD measurements.

### 2.3. Test Methods

Using an isothermal calorimeter (TAM Air, TA instruments, New Castle, DE, USA), the hydration heat characteristics (heat flow and heat of hydration) of the binders were measured by ASTM C 1702 [16]. Distilled water was used as a reference substance. To fabricate the paste required for the isothermal calorimetry measurement, the *w*/*b* ratio was set at 0.5. Subsequently, 5 g of paste was placed into a 20 mL glass sample vial, which was inserted into the isothermal calorimeter. The temperature was set at 23 °C, and the measurement was performed continuously for 3 days. To calculate the bound water and Ca(OH)_2_ contents of the binder with respect to the aging time, a TGA device (Thermo plus EVO2, Rigaku, Tokyo, Japan) was used. The reduction in weight was measured for the specimens that had been aged for 3, 7 and 28 days. The temperature range and heating conditions used for the TGA were 40–1000 °C and 10 °C/min, respectively. The bound-water and Ca(OH)_2_ contents were calculated according to the specimen weights at each temperature; these were determined using the TGA results and the following equations [17,18]:

(1) $$Bound\;water\left(\text{g/g}\right)=\frac{W_{40}-W_{480}}{W_{480}}$$

(2) $$Ca{\left(OH\right)}_{2}\left(\text{g/g}\right)=\frac{W_{400}-W_{480}}{W_{480}}\times \frac{74}{18}$$

where *Wn* is the dry sample weight at a temperature of *n* °C.

XRD (Miniflex 600, Rigaku, Tokyo, Japan) was employed to analyze the hydration product of each binder. Dry powder passed through a 75-μm sieve was used as the XRD specimen. Step scanning was conducted from 5°–65°, using a step interval of 0.02° and a scan speed of 1°/min. A pore-size distribution measurement was performed on the specimens that had been aged for 3, 7 and 28 days using porosimetry equipment (Autopore IV 9500, Micrometrics, Norcross, GA, USA). The surface tension and contact angle of the mercury used in the MIP measurements were 485 dynes/cm and 130°, respectively.

## 3. Results and discussion

### 3.1. Isothermal Calorimetry

**Figure 2:**
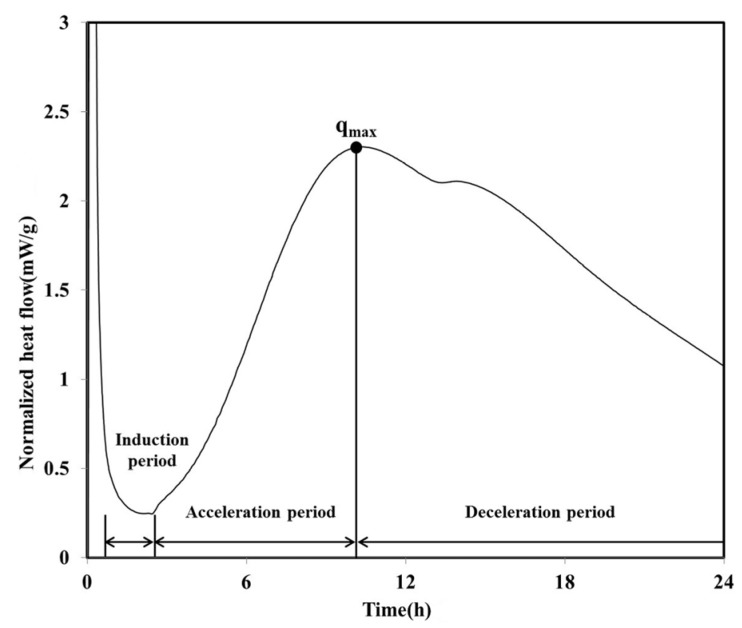
Normalized heat flow during initial 24 h of hydration (LH100).

The heat flow curve of the LH100 specimen, consisting of 100% LHC, exhibits a shape similar to that of OPC (Figure 2). Between approximately 1 and 2.5 h after the initiation of the hydration, an induction period was observed for the LH100 specimen. Following this induction period, the acceleration period commenced, in which hydration occurred; the maximum heat flow value (*q*_max_) of the LH100 specimen was 2.30 mW/g. 

**Figure 3:**
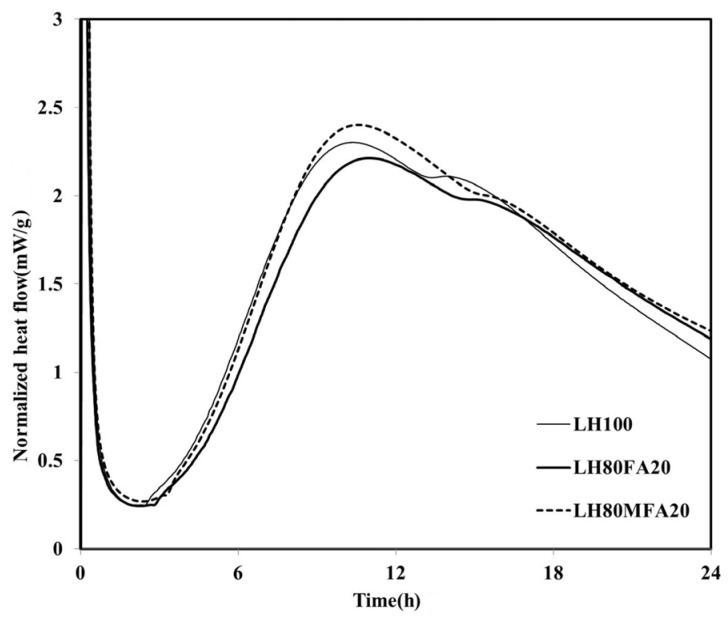
Normalized heat flows of LH80FA20 and LH80MFA20.

On reaching *q*_max_, the heat flow of the LH100 binder entered the deceleration period and subsequently decreased continuously. During the deceleration period, the amount of the calcium sulfate phase decreased, and a “shoulder” relating to the secondary aluminate reaction was observed [19]. The heat flows of the LH80FA20 and LH80MFA20 binders, in which 20 wt % of the LHC was substituted by FA and MFA of various levels of fineness, respectively, are shown in Figure 3. The LH80FA20 and LH80MFA20 binders had longer induction times than the LH100 binder; they also took a longer time to reach *q*_max_ and for the “shoulder” to appear. When FA was used as SCM, the delay phenomenon of early hydration (prior to 24 h) was exhibited because there was a decrease in the calcium concentration of the pore solution owing to the reaction of the aluminate with the calcium and FA in the pore solution; thus, the nucleation of the C–S–H was delayed [18]. However, the *q*_max_ values of the LH80FA20 and LH80MFA20 binders were higher than that of the LH100 binder, and following the appearance of the “shoulder”, the heat flow values were continuously higher than that of the LH100 specimen. The hydration of the cement was accelerated when FA was used as SCM because of the filler effect. When FA is used to replace cement, it fills the gaps between the cement particles. This yields additional nucleation sites, and because the effective ratio of water to cement increases, the hydration of the cement accelerates [20]. As shown in Figure 3, the *q*_max_ of the LH80MFA20 specimen (in which 20% MFA was used as SCM, which had a higher fineness than the FA, while having the same substitution ratio) was greater than that of the LH80FA20 specimen (in which FA with a low-fineness was used as SCM). The *q*_max_ value increased as the fineness of the FA increased because the increased fineness provided more nucleation sites, yielding a larger filler effect and the hydration reaction also accelerated due to the nucleation and filler effect [17,20,21,22].

**Figure 4:**
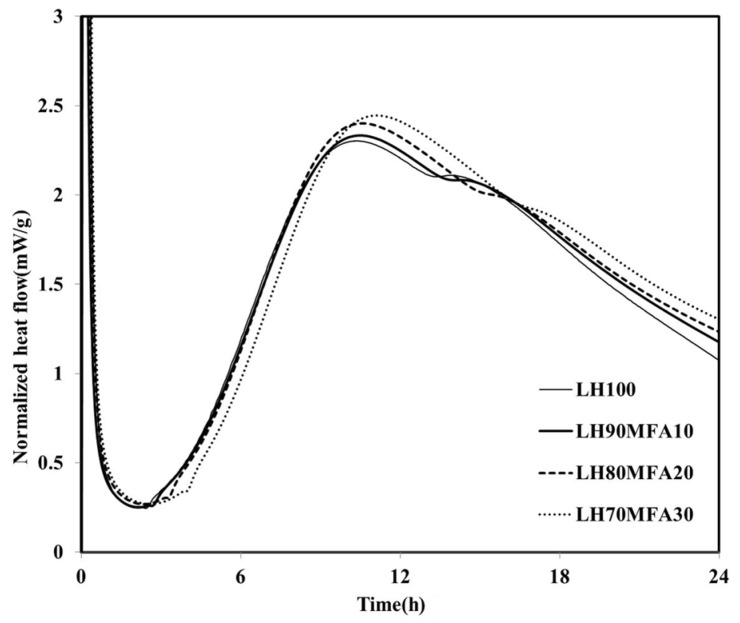
Normalized heat flows of LH90MFA10, LH80MFA20, and LH70MFA30.

**Figure 5:**
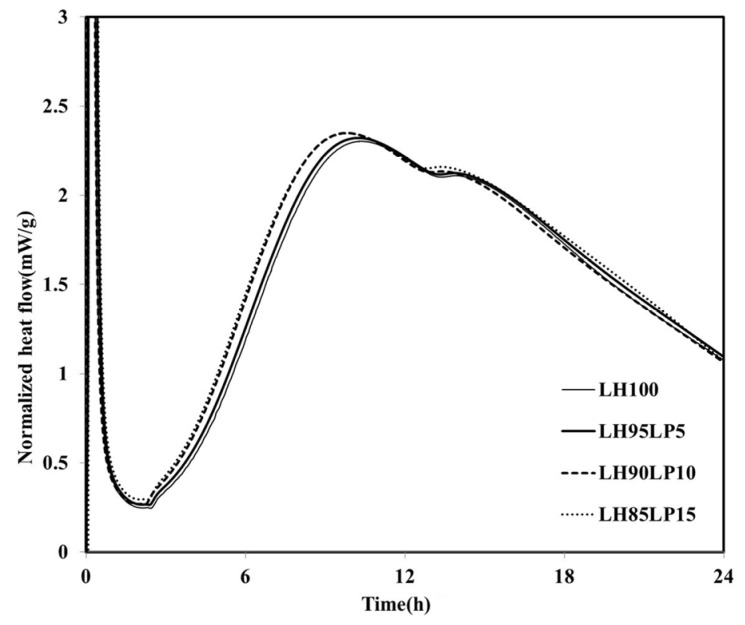
Normalized heat flows of LH95LP5, LH90LP10, and LH85LP15.

The heat flows of the specimens in which the LHC was substituted by 10%, 20% and 30% MFA (LH90MFA10, LH80MFA20 and LH70MFA30) are shown in Figure 4. As the proportion of MFA increased, the induction period increased, and there was a delay in the time taken for the “shoulder” to appear. With the increase in the amount of FA, the concentration of Ca in the pore solution of cement decreases, which delays the formation of C–S–H nuclei [23]. However, as the proportion of MFA increased, the hydration of the LHC accelerated; thus, between 17 and 48 h after the appearance of the “shoulder”, the *q*_max_ and heat flow values were high. As the proportion of the FA increased, the filler effect increased, and consequently, there was an acceleration in the hydration of the cement. When LP was used to replace the LHC (LH95LP5, LH90LP10 and LH85LP15), the sample demonstrated different heat-flow characteristics to those observed when FA was used as SCM (Figure 5). Compared with the LH100 binder, when LP was used as SCM, the heat flow values were neither increased nor delayed after reaching *q*_max_; the plotted results demonstrated curves almost identical to that of LH100. 

However, regardless of the LP to LHC ratio, compared to the results of the LH100 binder, there was an increase in the *q*_max_ and a reduction in the induction period, which is dissimilar to the case in which FA was used as SCM. The previous experimental investigations [17,18,23,24,25,26] showed that the induction period of cement tends to be shortened with the increase in the amount of limestone powder. This may be attributed to the fact that the formation of C–S–H nuclei can be accelerated by the physical absorption of CaCO_3_. By examining the hydration heat characteristics according to the amount of LP used as a substitute, the induction period decreased according to the increment of LP to LHC ratio (5%, 10% and 15%) and the *q*_max_ increased compared with those of LH100. 

**Figure 6:**
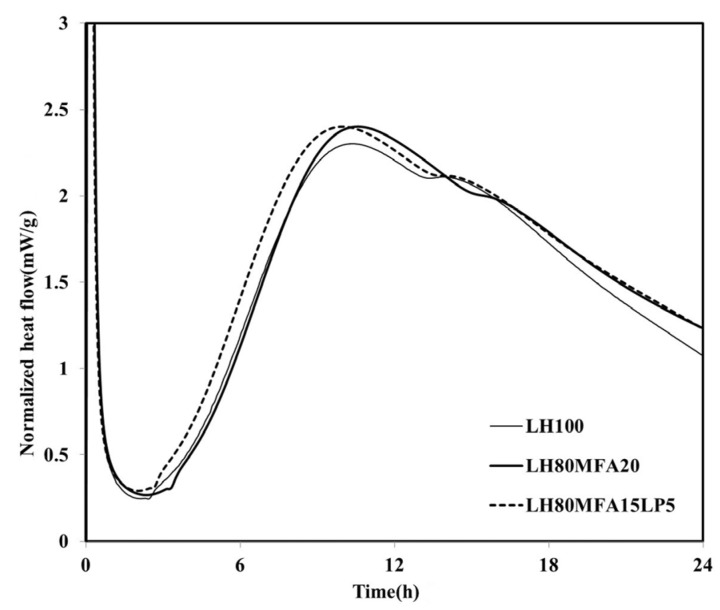
Normalized heat flow of LH80MFA15LP5.

Figure 6 shows the heat flow measurement results for the LH80MFA15LP5 specimen, a binder in which a mixture of FA and LP was used as SCM. The *q*_max_ of the LH80MFA15LP5 specimen was similar to that of the LH80MFA20 specimen, which used 20 wt % MFA as SCM for the LHC. However, the induction period, time taken to reach *q*_max_, and time at which the “shoulder” appeared for the LH80MFA15LP5 binder were lower than those for the LH80MFA20 binder. When the LHC was substituted by a mixture of FA and LP, the hydration-acceleration effect appeared to be more rapid than that when the LHC was replaced by FA alone.

Table 4 shows the 1-day and 3-day cumulative heat of hydration with respect to the binders used. For all the specimens, the cumulative heat of hydration increased as the aging time increased. The cumulative heat of hydration per g of LHC in the LH100 specimen was 129.50 and 192.01 J/g for 1 and 3 days of aging, respectively. Compared with the LH100 specimen, the cumulative heat of hydration per g of LHC was higher for the specimens in which the LHC was substituted by FA, LP and a mixture of FA and LP, regardless of the substitution ratio. Particularly, among the binders in which 20 wt % of the LHC was replaced, the LH80MFA15LP5 specimen (with a substitution mixture of FA and LP) exhibited a higher cumulative heat of hydration than the LH80FA20 and LH80MFA20 specimens, in which 20% of the LHC was replaced by FA alone.

However, the LH100 specimen had the highest cumulative heat of hydration per g of the binder because of the dilution effect. The substitution by the FA and LP reduces the content of LHC, which is a major reactant that produces heat and is directly involved in the hydration reaction [17].

**Table 4:** Cumulative heat of hydration emitted per g of low-heat cement (LHC) and per g of binder.

Binder	1 Day		3 Days	
	LHC (J/g)	Binder (J/g)	LHC (J/g)	Binder (J/g)
LH100	129.50	129.50	192.01	192.01
LH80FA20	129.71	103.77	193.76	155.01
LH90MFA10	130.88	117.79	196.56	176.90
LH80MFA20	133.01	106.41	207.23	165.78
LH70MFA30	133.76	93.63	215.30	150.71
LH95LP5	132.01	125.41	194.50	184.78
LH90LP10	133.21	119.89	193.96	174.56
LH85LP15	136.23	115.80	199.55	169.62
LH80MFA15LP5	137.70	110.16	209.30	167.44

### 3.2. TGA

**Figure 7:**
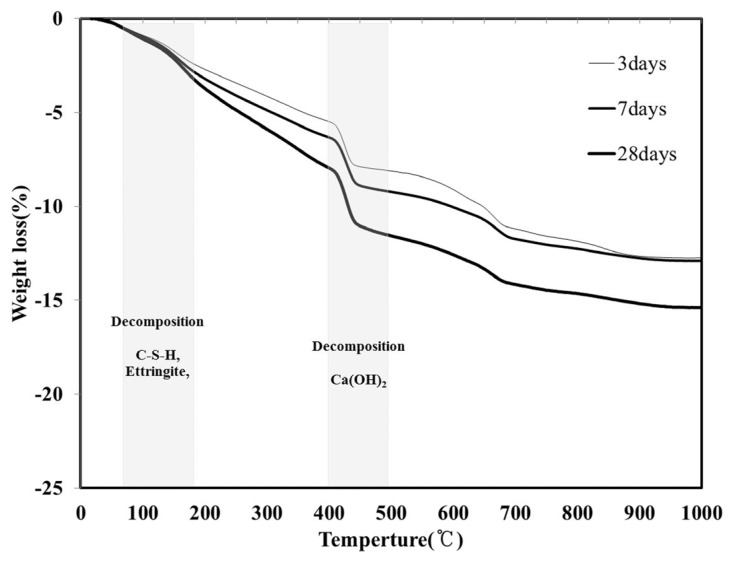
Thermogravimetric analysis (TGA) curve of the LH100 specimen at 3, 7 and 28 days.

In general, when a hydration reaction proceeds within a cement paste, hydration products such as C–S–H and ettringite are produced, the free water decreases, the bound water increases, and the amount of Ca(OH)_2_ increases. This is because the major mineral components of cement, *i.e.*, tricalcium silicate (C_3_S) and dicalcium silicate (C_2_S), produce C–S–H and Ca(OH)_2_ through a hydration reaction. Figure 7 shows the TGA results (following 3, 7 and 28 days) for the LH100 specimen, using 100% LHC. For all the aging conditions, the mass decreased because of the decomposition of hydration products such as C–S–H and ettringite in the temperature range of 70–180 °C. In the temperature range of 400–480 °C, a decrease in the mass due to the decomposition of another hydration product, Ca(OH)_2_, was observed. Because the amount of hydration product of the LH100 specimen increased with aging, there was also an increase in the mass reduction, as shown in Figure 7.

Table 5 shows the bound water per 100 g of LHC, as calculated for the LH100, LH80FA20, LH80MFA20 and LH80MFA15L5 specimens using the TGA measurement results. In all specimens, the amount of bound water increased with aging. For the specimens in which the LHC was substituted by FA and LP (LH80FA20, LH80MFA20 and LH80MFA15LP5), owing to the acceleration of the hydration, there was an increase in the bound water content compared with that of LH100, which used 100% LHC. 

**Figure 8:**
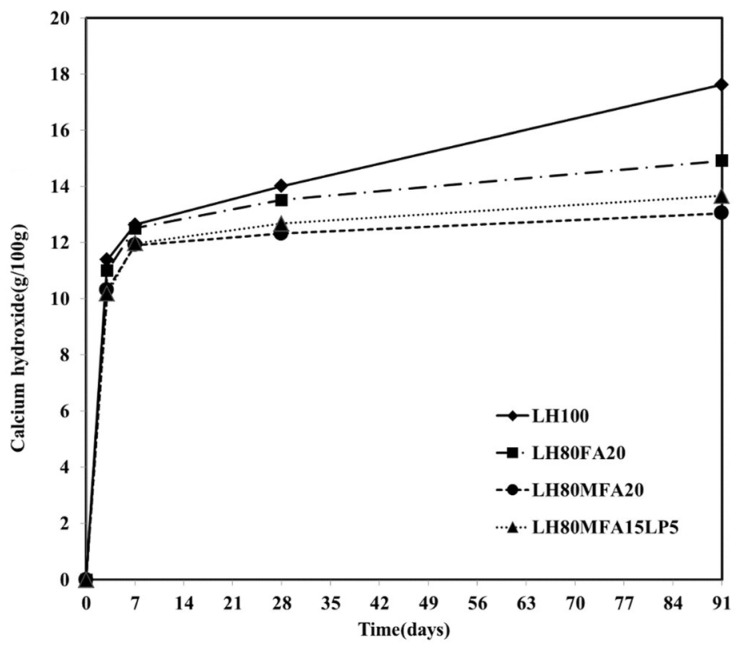
Ca(OH)_2_ content.

Figure 8 shows the Ca(OH)_2_ content by age for the LH100, LH80FA20, LH80MFA20 and LH80MFA15L5 specimens. The Ca(OH)_2_ contents were similar for all the specimens until an aging period of 7 days. However, following 28 days of aging, the Ca(OH)_2_ contents of the LH80FA20, LH80MFA20 and LH80MFA15L5 specimens were much lower than that of the LH100 specimen. Following 91 days of aging, the difference in the Ca(OH)_2_ content between these specimens was far larger. In the case of the LH100 specimen, the amount of Ca(OH)_2_ continuously increased with aging because of the hydration of the cement. However, when FA was used as SCM, the Ca(OH)_2_ was consumed by a pozzolanic reaction wherein additional C–S–H is formed because the Ca(OH)_2_ produced through the hydration of the cement reacts with the amorphous silicate of the FA [27,28]. The pozzolanic reaction of the FA was observed after 7 or 28 days of aging, and it densifies the internal structure of the cement paste and increases the long-term strength [29]. 

**Table 5:** Amount of bound water per 100 g of LHC.

Binder	3 days	7 days	28 days
	Bound Water (g)	Bound Water (g)	Bound Water (g)
LH100	8.60	9.86	12.22
LH80FA20	8.61	11.03	12.82
LH80MFA20	9.43	12.05	13.06
LH80MFA15LP5	8.75	11.02	12.23

Following 28 days of aging, the Ca(OH)_2_ content of the LH80MFA20 specimen with a high FA fineness is lower than that of the LH80FA20 specimen with a low FA fineness. Because the pozzolanic reactivity increases as the fineness of the FA increases, the Ca(OH)_2_ consumption also increases [17,20,30,31]. Following 28 days of aging of the LH80MFALP15 specimen (in which 15% MFA and 5% LP is used as SCM for 20% of the LHC), the Ca(OH)_2_ consumption was greater than that for the LH80FA20 specimen (in which 20% of the LHC is replaced with FA, which has a lower fineness than MFA) and lower than that for the LH80MFA20 specimen (in which 20% of the LHC is replaced with MFA). Specifically, following 28 days, the Ca(OH)_2_ was consumed because of the pozzolanic reaction of the MFA. However, the influence of LP on the Ca(OH)_2_ consumption was small.

### 3.3. XRD

**Figure 9:**
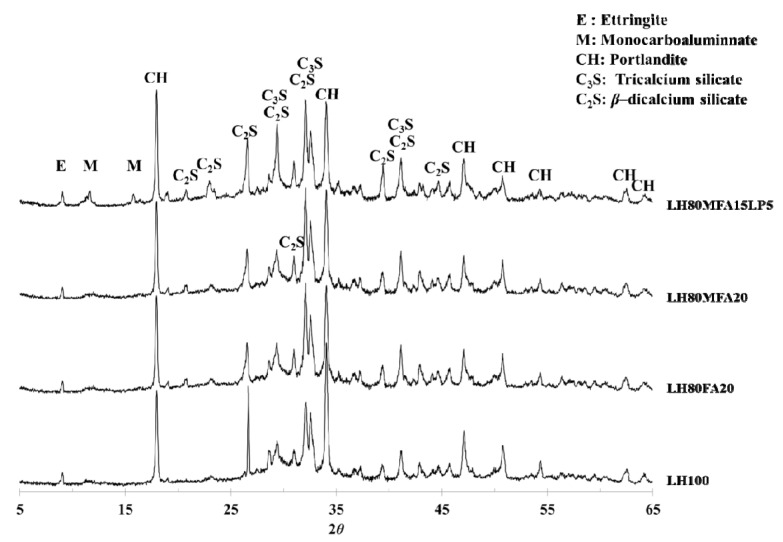
X-ray diffraction (XRD) patterns at 28 days.

Figure 9 shows XRD patterns of the LH100, LH80FA20, LH80MFA20 and LH80MFA15LP5 specimens at 28 days. In all the specimens, portlandite (Ca(OH)_2_) was observed as a major crystal phase. Ettringite and unhydrated C_2_S and C_3_S were also observed. C_3_A, a cement mineral, forms ettringite through a hydration reaction. However, if the amount of sulfate decreases within the cement paste, the ettringite reacts with the unhydrated C_3_A and produces monosulfate. In the case of such a conversion from ettringite to monosulfate, the volume of the hydration product decreases, and the porosity of the cement paste increases. However, if OPC is replaced by LP, the ettringite is not converted into monosulfate, and carboaluminate (hemicarboaluminate, monocarboaluminate) is produced [28,29,32].

LHC was used as the cement in this study. Compared with OPC, it has a lower C_3_A content and can thus form carboaluminate by reacting with CaCO_3_. However, monocarboaluminate was observed in the LH80MFA15LP5 specimen (in which a mixture of FA and LP was used as SCM), and this was not observed in the other specimens. This result correlates with the results of other researchers; when a mixture of FA and LP is used as SCM, alumina is supplied from the FA, and carboaluminate is subsequently formed [17,28].

### 3.4. MIP

**Figure 10:**
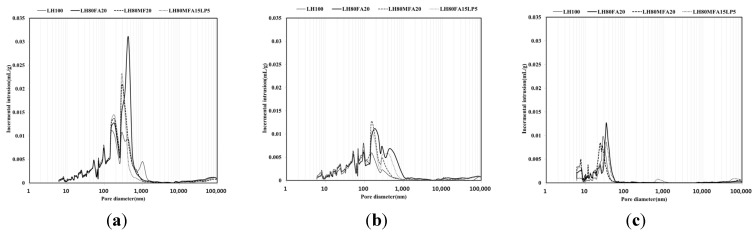
Pore-size distribution at: (**a**) 3 days; (**b**) 7 days; (**c**) 28 days.

Figure 10 shows the pore-size distribution for the LH100, LH80FA20, LH80MFA20 and LH80MFA15LP5 specimens with respect to age (3, 7 and 28 days). As the curing age increased, the porosity of each specimen decreased. At 3 and 7 days of ageing, macro-capillary pores of size 50–1000 nm were observed; however, following 28 days of aging, these macro-capillary pores were not observed. At 28 days of aging, the pores of each specimen consisted of micro-capillary pores of size 50 nm or smaller; the specimens that had been aged for 3 and 7 days had a similar amount of gel pores of size 10 nm or less. However, at 28 days, the specimens using binders in which FA was used as SCM (LH80FA20, LH80MFA20 and LH80MFA15LP5) had a larger amount of gel pores of size 10 nm or less than the specimen using LHC alone (LH100). At 28 days, the FA with a high fineness had a larger amount of gel pores than the FA with a low fineness. However, there was no increase in the amount of gel pores for the substitution mixture of FA and LP. 

As the aging progressed, the number of micropores inside the pastes increased, and the average pore size for each specimen decreased (Table 6). At 3 and 7 days of aging, the LH100 binder had a smaller average pore size than the specimens that used FA or LP as SCM (LH80FA20, LH80MFA20 and LH80MFA15LP5). However, at 28 days of aging, the specimens that used FA as SCM (LH80FA20, LH80MFA20 and LH80MFA15LP5) exhibited a smaller average pore size than that of the LH100 specimen. Additional hydrates were produced by the pozzolanic reaction of the FA, and the density of pores within the paste was high [20].

**Table 6:** Average pore size.

Binder	3 Days	7 Days	28 Days
	Average Pore Size (nm)	Average Pore Size (nm)	Average Pore Size (nm)
LH100	42.0	28.5	21.2
LH80FA20	49.7	38.1	18.5
LH80MFA20	49.1	32.9	15.0
LH80MFA15LP5	46.3	33.6	16.7

## 4. Conclusions

The heats of hydration, hydration products, and pore-size distribution of the proposed LHC-based binders partially replaced using FA, MFA and LP were examined to explore their potential application to mass concrete elements. The developed LHC-based binders need to be extended to evaluate the various properties of concrete, including setting behavior, compressive strength development, and durability. From the current experimental observations, the following conclusions may be drawn:

(1) In the case of using FA as SCM, the induction period and *q*_max_ arrival time at the hydration heat curve was delayed; however, the heat flow maintained a higher level than 100% LHC after *q*_max_ was reached. 

(2) In the case of using MFA, the induction period and *q*_max_ arrival time showed an identical curve characteristic to FA; however, as fineness and proportion of the FA increased, *q*_max_ per g of the LHC increased. 

(3) In the case of the type of additional LP, the induction period at the hydration heat curve shortened and *q*_max_ increased slightly as compared with the LHC-based binder.

(4) The hydration production of Ca(OH)_2_ was independent of the addition of FA or MFA up to an age of 7 days, beyond which the amount of Ca(OH)_2_ gradually decreased owing to their pozzolanic reaction. The addition of LP insignificantly affected the production of Ca(OH)_2_.

(5) The hydration products of LHC-based binder were insignificantly affected by the addition of FA or MFA, whereas the addition of LP generated monocarboaluminate as well.

(6) The average pore size measured at an age of 7 days was larger for LHC with FA or MFA than for 100% LHC; however, at an age of 28 days, smaller pore size was observed for LHC with FA or MFA than 100% LHC. As indicated, pozzolanic reaction of FA is commonly accelerated beyond an age of 7 days. As a result, the internal pore size of cement with FA gradually decreases with age.

## References

[1] Vance K., Aguayo M., Oey T., Sant G., Neithalath N. (2013). Hydration and strength development in ternary Portland cement blends containing limestone and fly ash or metakaolin. Cem. Concr. Compos..

[2] De Schutter G. (1999). Hydration and temperature development of concrete made with blast-furnace slag cement. Cem. Concr. Res..

[3] Zhang Y., Sun W., Liu S. (2002). Study on the hydration heat of binder paste in high-performance concrete. Cem. Concr. Res..

[4] Kumar A., Oey T., Falla G.P., Henkensiefken R., Neithalath N., Sant G. (2013). A comparison of intergrinding and blending limestone on reaction and strength evolution in cementitious materials. Constr. Build. Mater..

[5] Taylor H.F.W. (1997). Cement Chemistry.

[6] American Society for Testing and Materials (2015). Standard Specification for Portland Cement.

[7] BSI Standards Publication (2011). Cement. Composition, Specifications and Conformity Criteria for Common Cements.

[8] BSI Standards Publication (2015). Cement. Composition, Specifications and Conformity Criteria for very Low Heat Special Cements.

[9] Sasaki N., Goto T., Takao N., Naruse H. (2011). Improvement of thermal crack resistance using super low heat Portland cement whose belite is not less than 70 percent. Cem. Sci. Concr. Technol..

[10] Mori K., Fukunaga T., Sugiyama M., Iwase K., Oishi K., Yamamuro O. (2012). Hydration properties and compressive strength development of Low Heat Cement. J. Phys. Chem. Solids.

[11] Lee K.C., Cho J.W., Jung S.H., Kim J.H.J. (2011). Study on hydration heat of blended belite binder. J. Korea Concr. Inst..

[12] American Society for Testing and Materials (2015). Standard Test Methods for Chemical Analysis of Hydraulic Cement.

[13] American Society for Testing and Materials (2012). Standard Specification for Coal Fly Ash and Raw or Calcined Natural Pozzolan for Use in Concrete.

[14] American Society for Testing and Materials (2014). Standard Test Methods for Density of Hydraulic Cement.

[15] American Society for Testing and Materials (2011). Standard Test Methods for Fineness of Hydraulic Cement by Air-Permeability Apparatus.

[16] American Society for Testing and Materials (2015). Standard Test Methods for Measurement of Heat of Hydration of Hydraulic Cementitious Materials Using Isothermal Conduction Calorimetry.

[17] de Weerdt K., Sellevold E., Kjellsen K.O., Justnes H. (2011). Fly ash-limestone ternary cement: Effect of component fineness. Adv. Cem. Res..

[18] Deschner F., Winnefeld F., Lothenbach B., Seufert S., Schwesig P., Dittrich S., Goetz-Neunhoeffer F., Neubauer J. (2012). Hydration of Portland cement with high replacement by siliceous fly ash. Cem. Concr. Res..

[19] Gallucci E., Mathur P., Scrivener K. (2010). Microstructural development of early age hydration shells around cement grains. Cem. Concr. Res..

[20] Chindaprasirt P., Jaturapitakkul C., Sinsin T. (2007). Effect of fly ash fineness on microstructure of blended cement paste. Constr. Build. Mater..

[21] Zhang Y., Zhang X. (2008). Research on effect of limestone and gypsum on C_3_A, C_3_S and PC clinker system. Constr. Build. Mater..

[22] Bouasker M., Mounanga P., Turcruy P., Loukili A., Khelidj A. (2008). Chemical shrinkage of cement pastes and mortars at very early age: Effect of limestone filler and granular inclusions. Cem. Concr. Comp..

[23] Wang S., Chen C., Lu L., Cheng X. (2012). Effects of slag and limestone powder on the hydration and hardening process of alite-barium calcium sulphoaluminate cement. Constr. Build. Mater..

[24] Lothenbach B., Le Saout G., Gallucci E., Scrivener K. (2008). Influence of limestone on the hydration of Portland cements. Cem. Concr. Res..

[25] Thongsanitgarn P., Wongkeo W., Chaipanich A., Poon C.S. (2014). Heat of hydration of Portland high-calcium fly ash cement incorporating limestone powder: Effect of limestone particle size. Constr. Build. Mater..

[26] Péra J., Husson S., Guilhot B. (1999). Influence of finely ground limestone on cement hydration. Cem. Concr. Compos..

[27] Baert G., Hostel S., de Schutter G., de Belie N. (2008). Reactivity of fly ash in cement paste studied by means of thermogravimetry and isothermal calorimetry. J. Therm. Anal. Calorim..

[28] Lam L., Wong Y.L., Poon C.S. (2000). Degree of hydration and gel/space ratio of high volume fly ash/cement systems. Cem. Concr. Res..

[29] Sakai E., Miyahara S., Ohsawa S., Lee S.H., Daimon M. (2005). Hydration of fly ash cement. Cem. Concr. Res..

[30] Choi S.J., Lee S.S., Monteiro P.J.M. (2012). Effect of fly ash fineness on temperature rise, setting, and strength development of mortar. J. Mater. Civ. Eng..

[31] Kiattikomol K., Jaturapitakkul C., Songpiriyakij S., Chutubtim S. (2001). A study of ground coarse fly ashes with different finenesses from various sources as pozzolanic materials. Cem. Concr. Compos..

[32] Bonavetti V.L., Rahhal V.F., Irassar E.F. (2001). Studies on the carboaluminate formation in limestone filler-blended cements. Cem. Concr. Res..

